# Poster Session I - A47 COMPUTER-ASSISTED VERSUS UNASSISTED OPTICAL DIAGNOSIS OF COLORECTAL POLYPS: RESULTS FROM A LARGE PRAGMATIC IMPLEMENTATION STUDY

**DOI:** 10.1093/jcag/gwaf042.047

**Published:** 2026-02-13

**Authors:** M Oleksiw, E Medawar, D K Rex, C Hassan, R Djinbachian, V Michal, R Battat, E deslandres, M Bouin, J Liu, P Benoit, S Bouchard, D C Daoud, E Bernard, K Orlicka, S Sidani, R Leduc, H Sebajang, L D’aoust, D von Renteln

**Affiliations:** Centre Hospitalier de l’Universite de Montreal, Montreal, QC, Canada; Ottawa Hospital, Ottawa, ON, Canada; Indiana University School of Medicine, Indianapolis, IN; IRCCS Humanitas Research Hospital, Rozzano, Lombardia, Italy; Centre Hospitalier de l’Universite de Montreal, Montreal, QC, Canada; Centre Hospitalier de l’Universite de Montreal, Montreal, QC, Canada; Centre Hospitalier de l’Universite de Montreal, Montreal, QC, Canada; Centre Hospitalier de l’Universite de Montreal, Montreal, QC, Canada; Centre Hospitalier de l’Universite de Montreal, Montreal, QC, Canada; Centre Hospitalier de l’Universite de Montreal, Montreal, QC, Canada; Centre Hospitalier de l’Universite de Montreal, Montreal, QC, Canada; Centre Hospitalier de l’Universite de Montreal, Montreal, QC, Canada; Centre Hospitalier de l’Universite de Montreal, Montreal, QC, Canada; Centre Hospitalier de l’Universite de Montreal, Montreal, QC, Canada; Centre Hospitalier de l’Universite de Montreal, Montreal, QC, Canada; Centre Hospitalier de l’Universite de Montreal, Montreal, QC, Canada; Centre Hospitalier de l’Universite de Montreal, Montreal, QC, Canada; Centre Hospitalier de l’Universite de Montreal, Montreal, QC, Canada; Centre Hospitalier de l’Universite de Montreal, Montreal, QC, Canada; Centre Hospitalier de l’Universite de Montreal, Montreal, QC, Canada

## Abstract

**Background:**

Endoscopist optical diagnosis (OD) in real-time during colonoscopy could replace histopathological examination for diminutive (≤5mm) colorectal polyps. Computer-Aided Diagnosis (CADx) may help improve and standardize OD.

**Aims:**

We aimed to evaluate diagnostic performance of CADx-assisted OD versus unassisted OD for diminutive polyps encountered in routine clinical practice.

**Methods:**

We conducted a large prospective, IRB-approved, pragmatic implementation study (CER 22.013) between January 2021 and July 2025. Consecutive patients ≥40 years old undergoing elective colonoscopies were included. Procedures were performed with or without CADx assistance depending on CADx availability in the endoscopy suite and endoscopist choice. Our primary outcome was the sensitivity for adenoma diagnosis of CADx-assisted OD versus unassisted OD, using polyp histopathology diagnosis as ground truth.

**Results:**

A total of 1827 diminutive polyps, 860 with CADx diagnosis (of which 709 with CADx-assisted OD) and 979 without CADx use (CADx-unassisted OD), found in 966 patients (mean age 66 years, 46.8% female) were included. The polyp cohort consisted of 1120(61.3%) adenomas, 421(23.0%) hyperplastic polyps, 57(3.1%) sessile serrated lesions, and 212(12.3%) polyps with other histologies. 30 endoscopists, including 17 trainees, participated in the study. Adenoma sensitivity of CADx-assisted OD (85.6% [81.9-88.7]) did not differ compared to CADx-unassisted OD (83.8%, 95% CI: [80.7-86.5], p = 0.416, **Table 1**). However, adenoma sensitivity of the CADx output itself was significantly lower (76.1% [72.2-79.6]), p = 0.001. Adenoma specificity of CADx-assisted and unassisted OD were similar, p = 0.319. The rate of high confidence diagnoses was similar with or without CADx assistance, 84.9% (602/709) and 81.1% (794/979), respectively, and high confidence diagnoses in both groups had similar diagnostic performance.

**Conclusions:**

In this first large pragmatic implementation study reporting on the diagnostic performance of CADx-unassisted and CADx-assisted OD, there was no significant difference in endoscopist diagnostic performance with or without CADx assistance.

A47 Table 1: Diagnostic performance metrics (% (95% CI), n/N, p-value*) for CADx-Unassisted vs CADx-Assisted optical diagnoses, and vs CADx output

**Funding Agencies:**

CIHR, NoneTRIANGLE

**Table:** 

	CADx Unassisted	CADx Assisted	CADx Output
Accuracy	66.1% (63.1-68.9) 647/979	63.7% (60.2-66.9) 441/7090.271	61.4% (58.1-64.5) 525/8600.029
Sensitivity	83.8% (80.7-86.5) 515/615	85.6% (81.9-88.7) 347/4110.416	61.4% (58.1-64.5) 525/8600.001
Specificity	58.9% (53.9-63.7) 216/364	62.4% (57.1-67.4) 189/2980.319	64.6% (59.6-69.3) 230/3550.096
Positive Predictive Value	77.6% (74.4-80.5) 515/663	74.6% (71.1-77.8) 347/4560.180	74.6% (71.1-77.8) 384/5090.172
Negative Predictive Value	67.6% (62.8-72) 216/316	66.4% (62-70.6) 189/2530.671	64.8% (60.3-69.1) 230/3510.330

